# Electrical and optical properties of Al-doped ZnO and ZnAl_2_O_4_ films prepared by atomic layer deposition

**DOI:** 10.1186/1556-276X-8-144

**Published:** 2013-03-28

**Authors:** Qiongqiong Hou, Fanjie Meng, Jiaming Sun

**Affiliations:** 1 Key Laboratory of Weak Light Nonlinear Photonics, Ministry of Education, School of Physics, Nankai University, Weijin Road 94, Tianjin, 300071, China

**Keywords:** Aluminum-doped zinc oxide, Zinc aluminate, Atomic layer deposition, X-ray diffraction, Photoluminescence, 81.15.Gh, 72.20.Jv, 78.20.Ci

## Abstract

ZnO/Al_2_O_3_ multilayers were prepared by alternating atomic layer deposition (ALD) at 150°C using diethylzinc, trimethylaluminum, and water. The growth process, crystallinity, and electrical and optical properties of the multilayers were studied with a variety of the cycle ratios of ZnO and Al_2_O_3_ sublayers. Transparent conductive Al-doped ZnO films were prepared with the minimum resistivity of 2.4 × 10^−3^ Ω·cm at a low Al doping concentration of 2.26%. Photoluminescence spectroscopy in conjunction with X-ray diffraction analysis revealed that the thickness of ZnO sublayers plays an important role on the priority for selective crystallization of ZnAl_2_O_4_ and ZnO phases during high-temperature annealing ZnO/Al_2_O_3_ multilayers. It was found that pure ZnAl_2_O_4_ film was synthesized by annealing the specific composite film containing alternative monocycle of ZnO and Al_2_O_3_ sublayers, which could only be deposited precisely by utilizing ALD technology.

## Background

In the ZnO-Al_2_O_3_ composite material system, Al-doped zinc oxide (AZO) and zinc aluminate (ZnAl_2_O_4_) spinels are well known for their applications in optoelectronic devices and chemical industry. AZO was considered as an alternative low-cost transparent conductive oxide material instead of indium tin oxide in photovoltaic cells and displays [1,2]. ZnAl_2_O_4_ material has been used in many catalytic reactions, such as cracking, dehydration, hydrogenation, and dehydrogenation reactions [3,4]. As a wide-bandgap semiconductor material, ZnAl_2_O_4_ was also used as host of phosphors doping with Mn and rare earth ions [5,6]. AZO and ZnAl_2_O_4_ thin films have been deposited by different techniques [7], such as sol–gel coating [8], pulsed laser deposition [9], chemical vapor deposition [10], radio-frequency sputtering [11], and atomic layer deposition (ALD) [12,13]. Recently, ALD technology has been employed to grow transparent conductive AZO films with low resistivity in the order of 10^−3^ Ω·cm [14,15]. However, the correlation between the optical and the electrical properties in the ALD of AZO films has not yet been understood very well. Meanwhile, ZnAl_2_O_4_ film deposited on porous or nanostructure supporting materials by ALD technology may have large surface area and potential applications in catalysts and phosphors. However, since the ZnAl_2_O_4_ films need to be synthesized by annealing ZnO/Al_2_O_3_ composite films at elevated temperatures, the preferable crystallization of ZnO in the ALD of ZnO/Al_2_O_3_ composite films may strongly influence the purity of the synthesized ZnAl_2_O_4_ films. A detailed study on the correlation between the ZnO/Al_2_O_3_ cycle ratios in the multilayers and the formation of ZnO and ZnAl_2_O_4_ crystal phases during the subsequent thermal annealing would be crucial for synthesizing high purity ZnAl_2_O_4_ films.

In this paper, the ALD processes of the Al_2_O_3_ and ZnO thin films were studied using diethylzinc (DEZn), trimethylaluminum (TMA), and water with a variety of substrate temperatures. The growth temperature of the ZnO/Al_2_O_3_ composite films was determined by optimizing the growth temperature of ZnO layer according to the photoluminescence (PL) spectroscopy analysis. Then AZO films were prepared by adding a small fraction of Al_2_O_3_ doping cycles in the ALD process of ZnO films. The dependences of the crystalline structure, resistivity, and optical band gap of the AZO films on the Al doping concentration were studied in detail. Afterwards, multiple crystalline ZnAl_2_O_4_ films were synthesized by annealing the ALD ZnO/Al_2_O_3_ multilayers with a high fraction of Al_2_O_3_ layers. The influences of the ALD cycle ratio of the ZnO/Al_2_O_3_ sublayers and the annealing temperature on the formation of ZnO and ZnAl_2_O_4_ phases were studied by X-ray diffraction analysis. PL spectroscopy was used in conjunction with X-ray diffraction (XRD) to analyze traceable ZnO phase in thermal processed samples. It was found that pure ZnAl_2_O_4_ film was synthesized by annealing the specific composite film containing alternative monocycle of ZnO and Al_2_O_3_ sublayers, which could only be deposited precisely utilizing ALD technology.

## Methods

ZnO/Al_2_O_3_ composite films were deposited on quartz glass substrates or n-type Si substrates with (100) orientation. Before the film deposition, the Si substrates were cleaned through the Radio Corporation of America process, and the quartz glass substrates were treated by ultrasonic cleaning in alcohol and acetone. The ALD equipment is a 4-in. small chamber ALD system (Cambridge NanoTech Savannah 100, Cambridge NanoTech Inc., Cambridge, MA, USA). Diethylzinc (DEZn Zn(C_2_H_5_)_2_) and TMA Al(CH_3_)_3_ were used as the metal precursors for ZnO and Al_2_O_3_, respectively, while water vapor was used as oxidant. During the ALD process, the DEZn and TMA sources were not intentionally heated, and the precursor delivery lines were kept at 150°C. Nitrogen (99.999%) was used as carrier and purge gas with a flow rate of 20 sccm. One ZnO cycle consists of 0.015 s DEZn pulse time, 5 s N_2_ purge, 0.02 s H_2_O pulse time, and 5 s N_2_ purge. One Al_2_O_3_ cycle has 0.015 s TMA pulse time, 5 s N_2_ purge, 0.02 s H_2_O pulse time and 5 s N_2_ purge. First, pure ZnO and Al_2_O_3_ films were deposited on Si substrates with a variety of the growth temperature from 100°C to 350°C to determine the ALD windows. Then AZO films were deposited on quartz glass substrates at a temperature of 150°C. The total ALD cycles of ZnO plus Al_2_O_3_ layers are 1,090 for all the AZO samples, and the ALD cycles of the ZnO and Al_2_O_3_ sublayers in AZO films are varied with 50/1, 22/1, 20/1, 18/1, 16/1, 14/1, 12/1, and 10/1, respectively. For the ZnO/Al_2_O_3_ composite films with high fraction of Al_2_O_3_ sublayers, the total ALD cycles of the multilayers are 1,002, and the ALD cycles of the ZnO and Al_2_O_3_ sublayers are varied with 5/1, 4/1, 3/1, 2/1, 1/1, and 1/2, respectively. In order to synthesize crystalline ZnAl_2_O_4_ spinel films, the as-grown composite films were annealed subsequently in air at 400, 600, 700, 800, 1,000, and 1,100°C for 30 min, respectively.

The crystal structures of the samples were characterized by XRD analysis with Cu *K*_*α*_ radiation. The resistivity of the AZO films deposited on quartz substrate was measured using four-point probe technique. Transmission spectra were taken by a spectrometer with a 150 W Xe lamp. The thickness and the refractive index of the ZnO/Al_2_O_3_ composite films were measured by an ellipsometer with a 632.8-nm He-Ne laser beam at an incident angle of 69.8°. The average film growth per cycle was calculated by dividing the film thickness by the total number of ALD cycles. PL spectra from the films were measured at room temperature under the excitation of the 266 nm line of a Q-switch solid state laser (CryLas DX-Q; CryLaS GmbH, Berlin, Germany). The PL signal was collected by a 1/2-m monochromator and detected by a photomultiplier (model H7732-10) connected to a computer controlled Keithley 2010 multimeter (Keithley Instruments Inc., Cleveland, OH, USA). The topography of the ZnAl_2_O_4_ films was observed using a scanning electron microscope (SEM).

## Results and discussion

### Growth temperature of the ZnO/Al_2_O_3_ composite films

**Figure 1:**
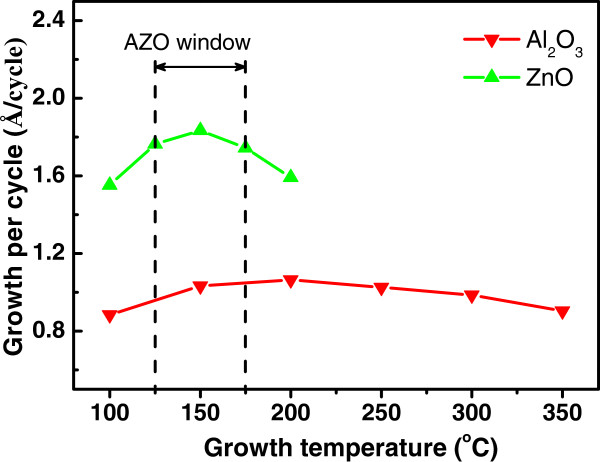
**Dependences of the growth per cycle of pure ZnO and Al**_**2**_**O**_**3 **_**films on the growth temperatures.**

In order to determine the common ALD growth temperature for ZnO/Al_2_O_3_ multilayers, the dependences of the growth per cycle on the substrate temperatures were studied on pure ZnO and Al_2_O_3_ films, respectively, as shown in Figure 1. The growth per cycle of the ZnO film increases from 1.55 to 1.83 Å as the deposition temperature increases from 100°C to 150°C, and then decreases to 1.59 Å as the temperature increases to 200°C, indicating a narrow ALD growth window of ZnO around 150°C with growth rate of 1.83 Å/cycle. The thermal dependence of the growth rate of Al_2_O_3_ shows a nearly constant value at around 1.0 Å/cycle in a wide temperature window from 100°C to 350°C. The optimized growth temperature for growth of uniform ZnO/Al_2_O_3_ multilayer should be optimized within the overlap region of the two ALD windows. Optimization should be done according to the growth temperature for high-quality ZnO films.

**Figure 2:**
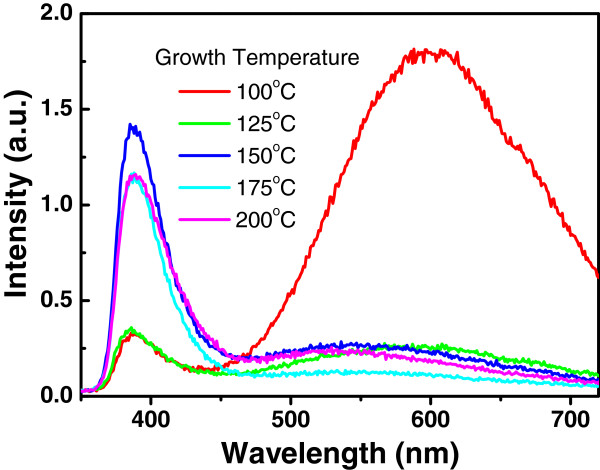
Room temperature PL spectra from the ZnO films with different growth temperatures.

The crystal quality of a semiconductor is normally evaluated by the efficiency of its band-edge photoluminescence; therefore, room temperature PL spectra of the ZnO films grown at different temperature were studied under the excitation of a 266-nm laser, as shown in Figure 2. The ultraviolet (UV) peak at around 387 nm is from the near-band-edge emission of crystalline ZnO, while the broad peak around 600 nm can be ascribed to the radiative recombination at the defects in ZnO films. The intensity of the UV peak increases with increasing the growth temperature from 100°C to 150°C, with a maximum growth temperature at 150°C and saturation at higher growth temperature up to 200°C. In the meantime, the luminescent band at 600 nm from the defects strongly decreases from 100°C to 150°C. This indicates that the crystalline quality of the ZnO film is getting better with a decrease of the defect density from 100°C to 150°C and become stable at higher growth temperatures up to 200°C. Luca et al. [16] reports an increase of the PL intensity with further increasing growth temperature from 200°C to 240°C, indicating a better crystal quality of the ZnO film at higher growth temperature. However, ZnO films cannot be deposited uniformly in ALD mode at higher temperatures above 200°C due to the thermal decomposition of DEZn precursor [17]. As a consequence, the optimized growth temperature for deposition of ZnO/Al_2_O_3_ composite films was selected at 150°C. The growth rates of the pure ZnO and Al_2_O_3_ films were 1.83 and 1.03 Å per cycle at this temperature, respectively, which are consistent with the reported values in [18].

### The AZO films

AZO films with overall 1,090 cycles of ZnO plus Al_2_O_3_ layers were alternatively deposited on quartz substrates at 150°C. The ALD cycles in the ZnO/Al_2_O_3_ supercycles are 50/1, 22/1, 20/1, 18/1, 16/1, 14/1, 12/1, and 10/1, where monocycle Al_2_O_3_ doping layers were inserted between different cycles of ZnO sublayers. Since the real Al concentration matches the ‘rule of mixtures’ formula well at lower Al concentration below 5%, in which the growth rate of the AZO is close to pure ZnO [19]. The Al concentration in the AZO films was calculated using the following formula:

(1) $$C_{\mathrm{Al}}=\frac{\rho_{\mathrm{Al}}C_{{\mathrm{Al}}_{2}O_{3}}}{\rho_{\mathrm{Al}}C_{{\mathrm{Al}}_{2}O_{3}}+\rho_{\mathrm{Zn}}\left(1-C_{{\mathrm{Al}}_{2}O_{3}}\right)},$$

where $C_{Al_{2}O_{3}}$ is the percentage of Al_2_O_3_ cycles, *ρ*_Al,_ and *ρ*_Zn_ are the densities of Al and Zn atoms deposited during each ALD cycle for the pure Al_2_O_3_ and ZnO films, respectively. The densities of Al_2_O_3_ and ZnO growth by ALD are 2.91 and 5.62 g/cm^3^[20], So *ρ*_Al_ and *ρ*_Zn_ were calculated to be 5.89 × 10^−10^ mol/cm^2^/cycle and 1.27 × 10^−9^ mol/cm^2^/cycle, respectively.

**Figure 3:**
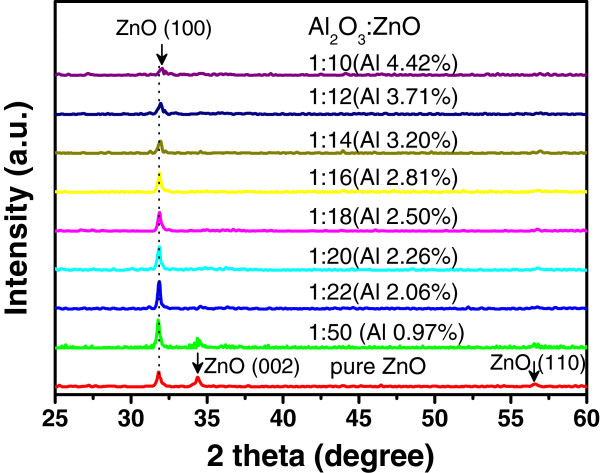
XRD patterns of the AZO films with different Al content from 0% to 4.42%.

Figure 3 shows the XRD patterns of the AZO films grown on quartz substrate with different ZnO/Al_2_O_3_ cycle ratios that are varied from 50:1 to 10:1 (corresponding to Al concentration from 0.96% to 4.42%). The diffraction pattern of the pure ZnO film without Al_2_O_3_ doping layer is also shown as a reference. The X-ray diffraction pattern from pure ZnO film exhibits multiple crystalline ZnO structure with (100), (002), and (110) peaks [17]. With increasing the Al doping concentration, the (002) and (110) diffraction peaks decrease strongly, thus the AZO films exhibiting (100) dominated the orientation. The intensity of the (100) diffraction peak reaches a maximum at 2.06% (with the ratio of ZnO/Al_2_O_3_ layers is 22/1), and then it decreases at higher Al concentration above 3%. The preferred (100) orientation of the AZO films in our samples is consistent with the results reported by Banerjee et al. [18]. It is worthy to note that the Al_2_O_3_ layer by ALD is amorphous at the growth temperature of 150°C, so the decrease of the (100) peak at higher Al concentration can be explained that the amorphous Al_2_O_3_ doping layers destroy the crystal quality during the growth of AZO films. Figure 3 also shows that the (100) peak of ZnO shifts to larger diffraction angle with increasing the concentration of Al in AZO films. This can be interpreted as that the increase of the Al concentration will reduce the lattice constant by substitutions of Zn^2+^ ions (ion radius 0.74 Å) with smaller Al^3+^ (0.53 Å) ions; therefore, the (100) peak of ZnO shifts to larger diffraction angle in AZO films.

**Figure 4:**
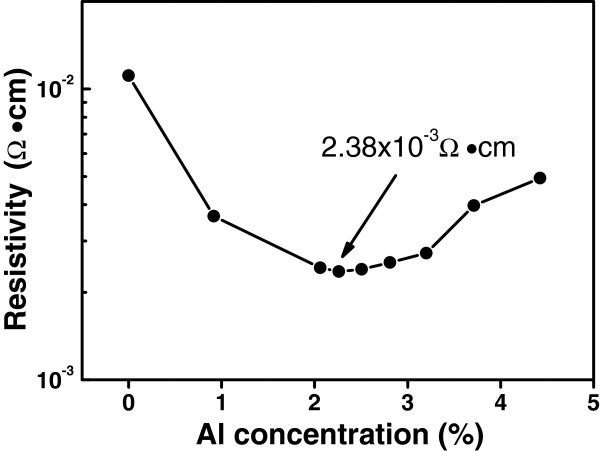
Dependence of the resistivity of AZO films on Al concentration.

Figure 4 plots the resistivity of AZO films as a function of Al concentration, which was measured by four-point probe technique. As the Al concentration increases from 0% to 2.26%, the resistivity initially decreases from 1.11 × 10^−2^ to a minimum of 2.38 × 10^−3^ Ω·cm, and then increases at higher Al doping concentration. This result is comparable to the values of the ALD grown AZO films reported by other groups [12,14,21]. The decreasing of the resistivity may attribute to the increase of Al donor concentration by substitution of Zn^2+^ sites with Al^3+^ ions in the ZnO lattices. However, it should be noted that the variety of resistivity in Figure 4 is also in strong correlation to the change of crystal quality in the AZO films at different Al doping concentrations, as shown in Figure 3. Initially, the decrease of the resistivity with increasing the Al concentration from 0% to 2.26% is related to the improvement of the crystal quality of the AZO films, as it was indicated by the increased intensity of the (100) X-ray diffraction peak in Figure 3. The AZO film with the best crystal quality has the minimum resistivity of 2.38 × 10^−3^ Ω·cm at Al concentration of 2.26%. At higher Al doping concentration above 3%, a decrease of the intensity of the (100) diffraction peak indicates a degeneration of the crystal quality; as a consequence, an increase of the resistivity was shown in Figure 4. The reason for the increase of the resistivity at high Al concentration is probably related to the formation of Zn vacancy acceptors or the formation of homologous phase like ZnAl_*x*_O_*y*_ or Al_2_O_3_ in the AZO films [9,22].

**Figure 5:**
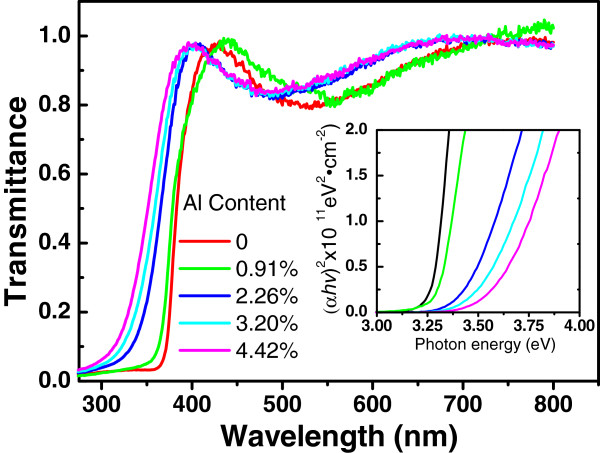
**Transmission spectra of AZO films deposited on quartz glasses.** The inset is the plots of (*α*hν)^2^ versus photon energy.

**Figure 6:**
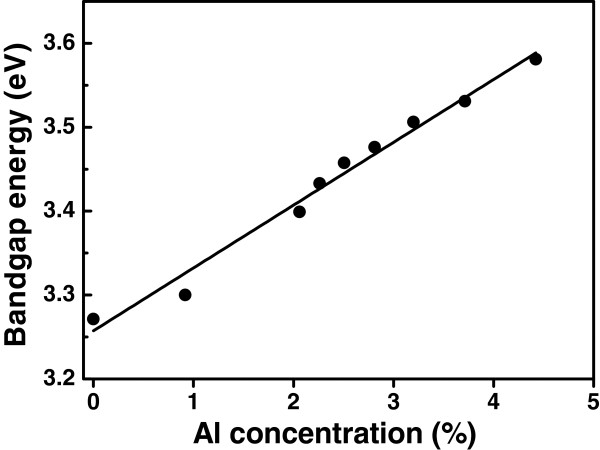
Dependence of the band gap energy of AZO films on Al concentration.

The transmission spectra of the AZO films deposited on quartz glasses are shown in Figure 5. The average transmittance was above 80% in the visible wavelength, regardless of the Al concentration in the AZO films. A blue shift of the optical band edge was observed with increasing the Al concentration. The relationship between absorption coefficient and optic band gap of direct band gap semiconductor is given by Tauc equation [23], (*α*hv)^2^ = *B*(hv − *E*_g_), where α is the absorption coefficient, hν is the photon energy, *B* is a constant, and *E*_g_ is the optical band gap energy, respectively. The dependence of *(α*hν*)*^2^ on photon energy was plotted in the inset of Figure 5. The band gap energy was obtained by the extrapolations of the liner regions of the optical absorption edges. Figure 6 shows the variation of band gap energy versus Al concentration. The band gap energy increased from 3.27 to 3.58 eV with increasing Al concentration from 0% to 4.42%. A linear fit to the bandgap energy versus Al concentration gives *E*_g_*=* 3.26 *+* 0.0749*x*_Al_, where *E*_g_ is the band gap energy of AZO, *x*_Al_ is the Al concentration of AZO. The correlation between the blue shift of the absorption edge and the increased conductivity with Al doping can be attributed to the Bustein-Moss increase of the band gap with increasing carrier concentration in semiconductors [12].

**Figure 7:**
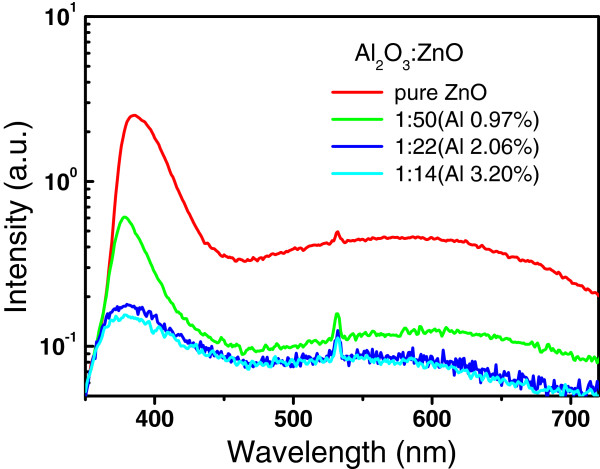
Room temperature PL spectra excited by a 266-nm laser for AZO films with different Al concentration.

Figure 7 shows the room temperature PL spectra excited by a 266-nm laser for AZO films with different Al concentrations. The blue shift of the UV peaks from the near-band-edge emission of ZnO is consistent with the results from the transmittance spectra in Figure 5 and Figure 6. The intensity of the PL decreases strongly with increase of the Al concentration from 0% to 3.2% in the as-prepared AZO films. This is probably due to the introduction of the nonradiative recombination centers with increasing fraction of the amorphous Al_2_O_3_ doping layers in AZO films.

### ZnAl_2_O_4_ films

**Figure 8:**
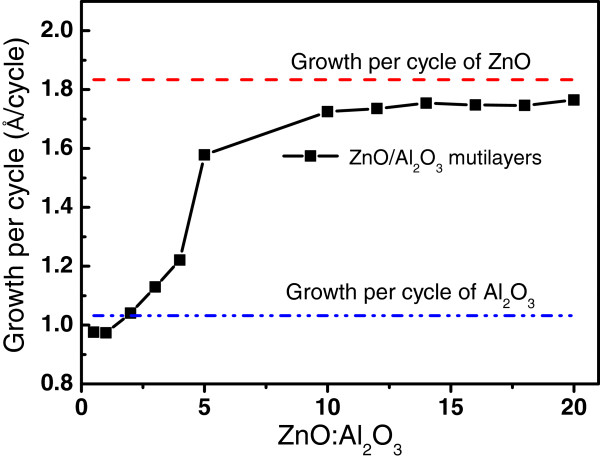
**Dependence of the growth per cycle of the ZnO/Al**_**2**_**O**_**3 **_**composite films on the ZnO/Al**_**2**_**O**_**3 **_**cycle ratio.**

Starting ZnO/Al_2_O_3_ composite films with high fraction of Al_2_O_3_ layers were grown by ALD prior to synthesis of the ZnAl_2_O_4_ films by high temperature annealing process. Figure 8 shows the dependence of the average growth per cycle on the ZnO/Al_2_O_3_ cycle ratio in the multilayers. The average growth per cycle of the composite films at ZnO/Al_2_O_3_ ratio of 1:2 and 1:1 is smaller than the growth rate of pure ZnO and Al_2_O_3_ layers. The reason is that there is a strong etching of the pre-deposited ZnO layer during exposure ZnO surface to the TMA precursor in the ALD cycle of Al_2_O_3_, as discussed in detail in [18,19]. The removal of the ZnO surface layer causes a reduction of average growth rate especially when the thickness of the ZnO sublayers reduces to several cycles. The influence of the surface etching of ZnO sublayer on the growth rate can be eliminated by increasing the thickness of the ZnO sublayer. This is observed by the strong increase of the average growth per cycle with increasing ZnO sublayer thickness from 1 to 10 cycles in Figure 8. The average growth rate is almost constant at around 1.75 Å/cycle during the ALD ZnO/Al_2_O_3_ multilayers when the ALD cycles of the ZnO/Al_2_O_3_ sublayers is above 10:1, which is close to the growth rate of pure ZnO (1.838 Å/cycle).

**Figure 9:**
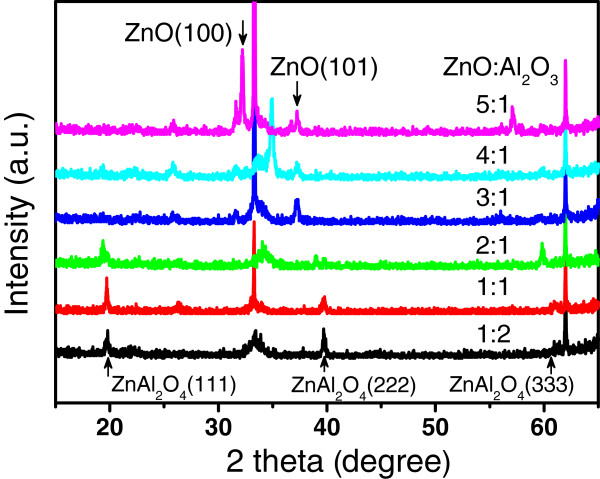
**XRD patterns of the compound films at different ZnO/Al**_**2**_**O**_**3 **_**cycle ratios.**

Attention has been paid to select the starting specific ZnO/Al_2_O_3_ composite films with appropriate sublayer thicknesses for synthesizing pure ZnAl_2_O_4_ films. ZnO/Al_2_O_3_ multilayers with different ZnO/Al_2_O_3_ cycle ratios from 1:2 to 5:1 were grown by ALD and then subsequently annealed at 1,000°C for 0.5 h. Figure 9 shows the XRD patterns of the annealed samples with different ZnO/Al_2_O_3_ cycle ratios. The XRD patterns of the annealed composite films show (111), (222), and (333) peaks of ZnAl_2_O_4_ spinel structure for the ZnO/Al_2_O_3_ cycle ratios at 2:1, 1:1, and 1:2 respectively, indicating that only ZnAl_2_O_4_ films with spinel crystal structure are synthesized from these specific ZnO/Al_2_O_3_ starting multilayers by ALD. A competition process of the easy ZnO crystallization with the formation of crystalline ZnAl_2_O_4_ is observed with the increasing thickness of ZnO sublayer. It should be noted that only the (100) and (101) peaks of the hexagonal phase ZnO appear and no crystalline Al_2_O_3_ or ZnAl_2_O_4_ peaks are detectable in the X-ray diffraction patterns of the samples with cycle ratio of 3:1, 4:1, and 5:1, where the ZnO sublayer thickness is more than three ALD cycles. This reveals that the thickness of the ZnO sublayer in the ZnO/Al_2_O_3_ composite films is a crucial parameter for the control of the formation of ZnO and ZnAl_2_O_4_ phases during the thermal annealing process. Taking into account of the etching during the Al_2_O_3_ cycle, the measured ZnO sublayer thickness is 0.91 and 2.01 Å in the samples with the ZnO/Al_2_O_3_ cycle ratios of 2:1 and 1:1. Comparing to the reported length of the Zn-O bond (1.98 Å) [24], the critical thickness of the ZnO sublayer is limited within one atomic layer for the formation of the ZnAl_2_O_4_ phase. This can be interpreted by the chemical reaction for synthesis of the ZnAl_2_O_4_, ZnO + Al_2_O_3_ = ZnAl_2_O_4_, where one monolayer of Al_2_O_3_ consumes one atomic layer of ZnO. Thicker ZnO sublayer containing excess atomic layers has a priority forming in the ZnO crystal phase of the annealed ZnO/Al_2_O_3_ multilayers, because the crystallization of ZnO need much lower energy than that for the ZnAl_2_O_4_ crystallization.

**Figure 10:**
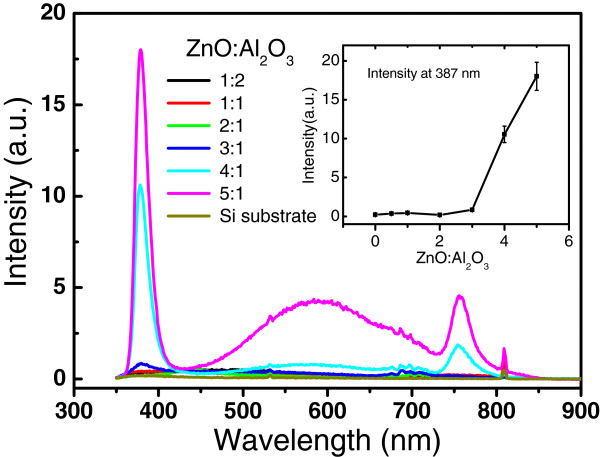
**Room temperature PL spectra of the ZnO/Al**_**2**_**O**_**3 **_**composite films with different ZnO/Al**_**2**_**O**_**3 **_**cycle ratios.**

Room temperature PL spectroscopy was used to analyze and control traceable amount of the crystalline ZnO phase in the annealed samples. Figure 10 shows the PL spectra from the ZnO/Al_2_O_3_ mutilayers annealed at 1,000°C with different cycle ratios of ZnO/Al_2_O_3_ from 1:2 to 5:1. No PL signal from the crystalline ZnO is observed for the annealed samples with the ZnO/Al_2_O_3_ cycle ratios at 2:1, 1:1, and 1:2, respectively; this is supported by the XRD results in Figure 9, which showed only diffraction peaks of spinel ZnAl_2_O_4_ without ZnO impurity phase in these samples. The PL intensity from ZnO near-band-edge emission increases strongly as the ZnO sublayer thickness increases above three ALD cycles; this is also in good agreement with the formation of ZnO phase in the samples with ZnO/Al_2_O_3_ ratios of 3:1 to 5:1. These results reveal that the presence of excess ZnO bonds leads to the formation of the ZnO crystal phase due to the easy crystallization of ZnO. The specific multilayers containing alternative monatomic layers of ZnO and Al_2_O_3_ are crucial as the starting composite for synthesis of pure ZnAl_2_O_4_ films. The composite can only be deposited precisely through layer by layer ALD technology. Preformation of Zn-O-Al-O bonds at the interface of two ZnO/Al_2_O_3_ multilayers during the ALD process may play an important role for the crystallization of pure ZnAl_2_O_4_ films in the subsequent high-temperature annealing.

**Figure 11:**
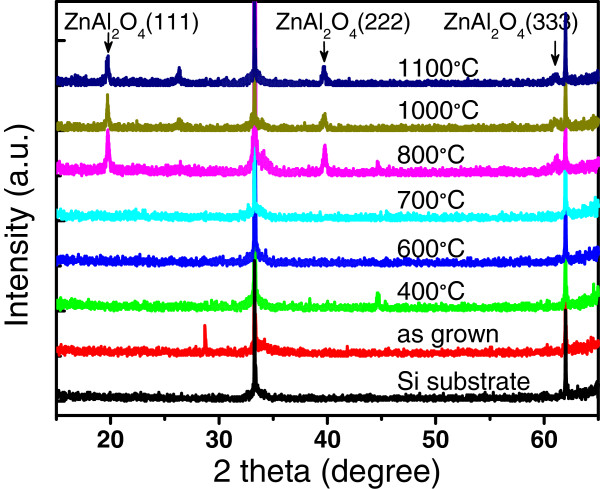
**XRD spectra of the ZnO/Al**_**2**_**O**_**3 **_**composite films after annealed at different temperatures.**

**Figure 12:**
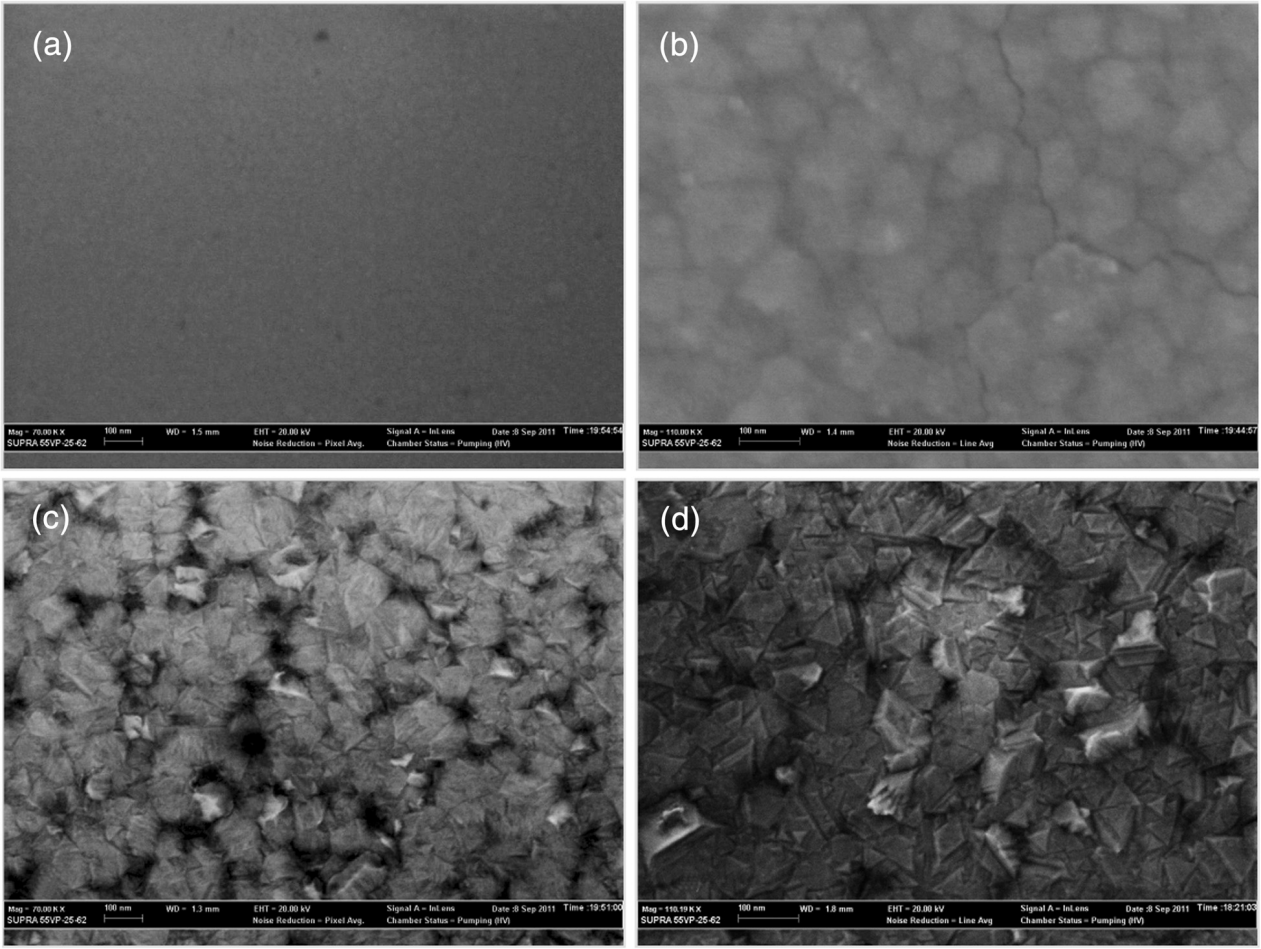
**SEM images of the ZnO/Al**_**2**_**O**_**3 **_**composite films with optimized ZnO/Al**_**2**_**O**_**3 **_**monocycle ratio of 1:1.** Samples were annealed at 700°C (**a**), 800°C (**b**), 1,000°C (**c**), and 1,100°C (**d**), respectively.

Figure 11 shows the XRD patterns of the composite films after annealed at different temperatures ranging from 400 to 1,100°C, in which the ZnO/Al_2_O_3_ cycle ratio of the composite film was set to 1:1. The XRD patterns of the as-grown ZnO/Al_2_O_3_ composite film and those annealed below 800°C show amorphous layers, where no diffraction peak from ZnAl_2_O_4_ crystal was observed. When the annealing temperature is above 800°C, diffraction peaks of (111), (222), and (333) from the cubic phase of the ZnAl_2_O_4_ spinel structure appear in the XRD patterns. This result shows that the multiple crystalline ZnAl_2_O_4_ film is synthesized by the high temperature annealing process above 800°C. The surface morphologies of the samples annealed at different temperatures of 700, 800, 1,000, and 1,100°C were observed by SEM, as shown in Figure 12a,b,c,d. The film annealed at relatively low temperature of 700°C for 0.5 h had a smooth surface morphology as shown in Figure 12a. At annealing temperature of 800°C, the film starts to crystallize, with significant grain boundaries emerge on the surface, as shown in Figure 12b. The crystalline grains in the film grow up with increasing annealing temperature from 1,000 to 1,100°C, as shown in Figure 12c,d.

## Conclusions

AZO and ZnAl_2_O_4_ films were prepared by alternating atomic layer deposition (ALD) of ZnO/Al_2_O_3_ laminates using DEZn, TMA and water. A deposition temperature of 150°C was selected for the ZnO/Al_2_O_3_ composite films. The growth per cycle, structure, electrical, and optical properties of the ZnO/Al_2_O_3_ laminates were studied at different Al concentration, which was controlled by varying the cycle ratio of ZnO/Al_2_O_3_ from 1:2 to 50:1. It is shown that the growth rate of the ZnO is reduced during the ALD of ZnO/Al_2_O_3_ multilayers due to the etching of the ZnO surface layer during exposure to TMA precursor in Al_2_O_3_ cycle. Conductive transparent AZO films were obtained at low Al doping concentration with the minimum resistivity of 2.38 × 10^−3^ Ω·cm and transmittance above 80% in the visible range. The PL spectroscopy in conjunction with XRD reveals that pure ZnAl_2_O_4_ film was synthesized from the composite with alternative monocycle of ZnO and Al_2_O_3_ deposited by precise ALD technology. SEM and XRD studies indicate that the crystalline ZnAl_2_O_4_ films can be synthesized at annealing temperature from 800°C to 1,100°C.

## Abbreviations

ALD: atomic layer deposition; AZO: Al-doped ZnO; DEZn: diethylzinc; PL: photoluminescence; SEM: scanning electron microscope; TMA: trimethylaluminum; XRD: X-ray diffraction; UV: ultraviolet

## Competing interests

The authors declare that they have no competing interests.

## Authors’ contributions

QQH performed the experiment of the ZnAl_2_O_4_ films and drafted the manuscript. FJM performed the experiment of the pure ZnO, Al_2_O_3,_ and AZO films. JMS carried out the designation and the preparation of the study, supervised the work, and finalized the manuscript. All authors read and approved the final manuscript.
